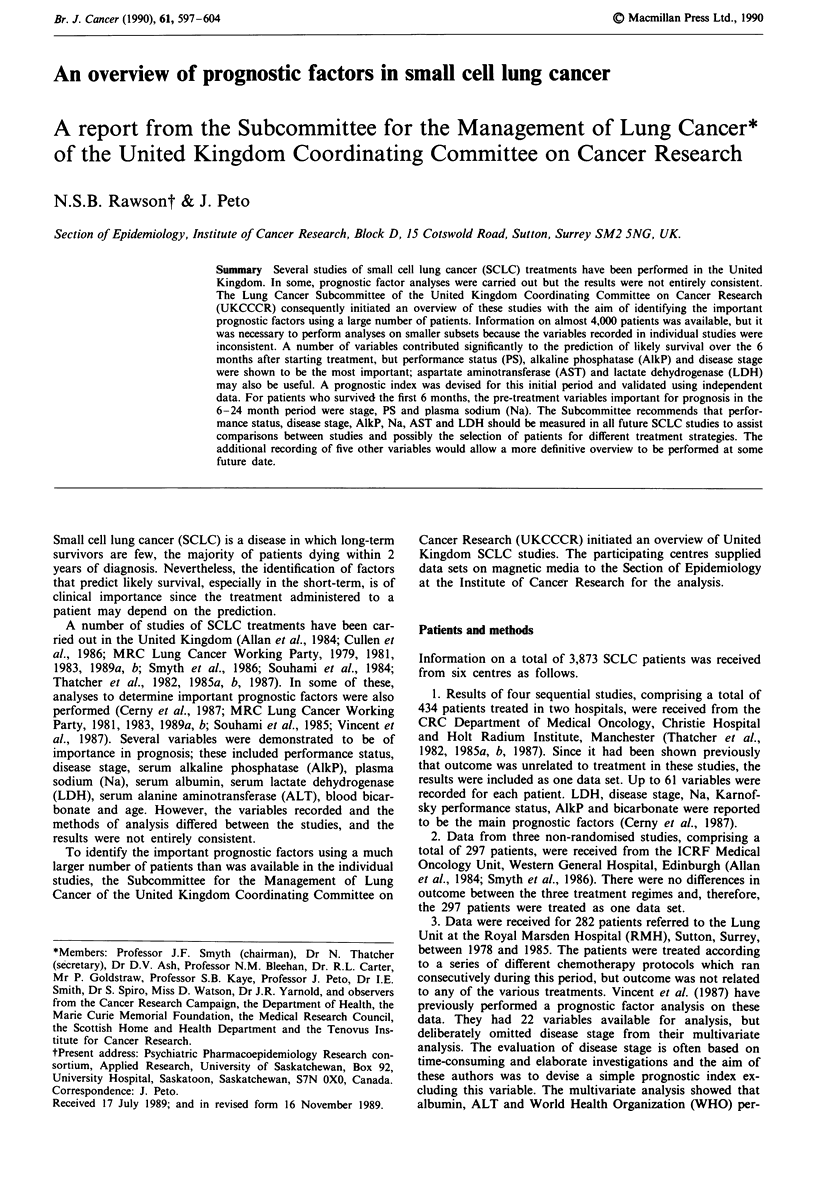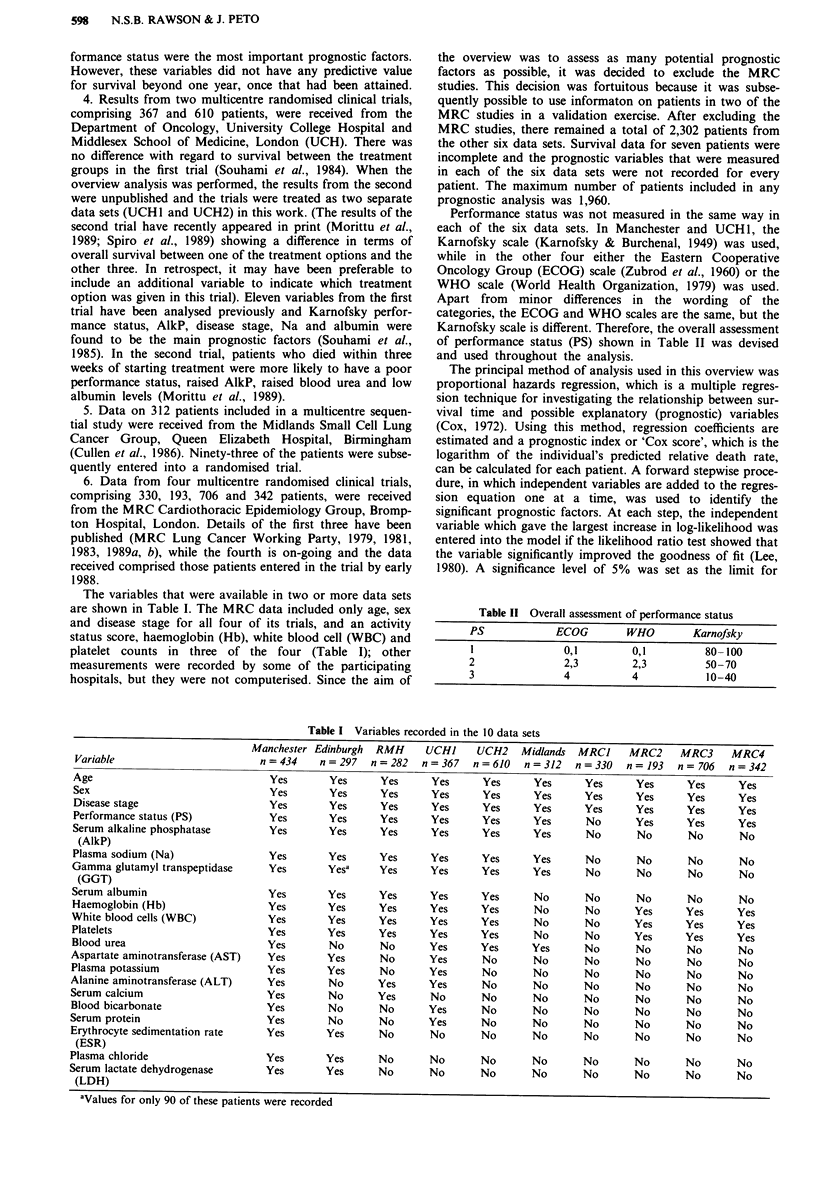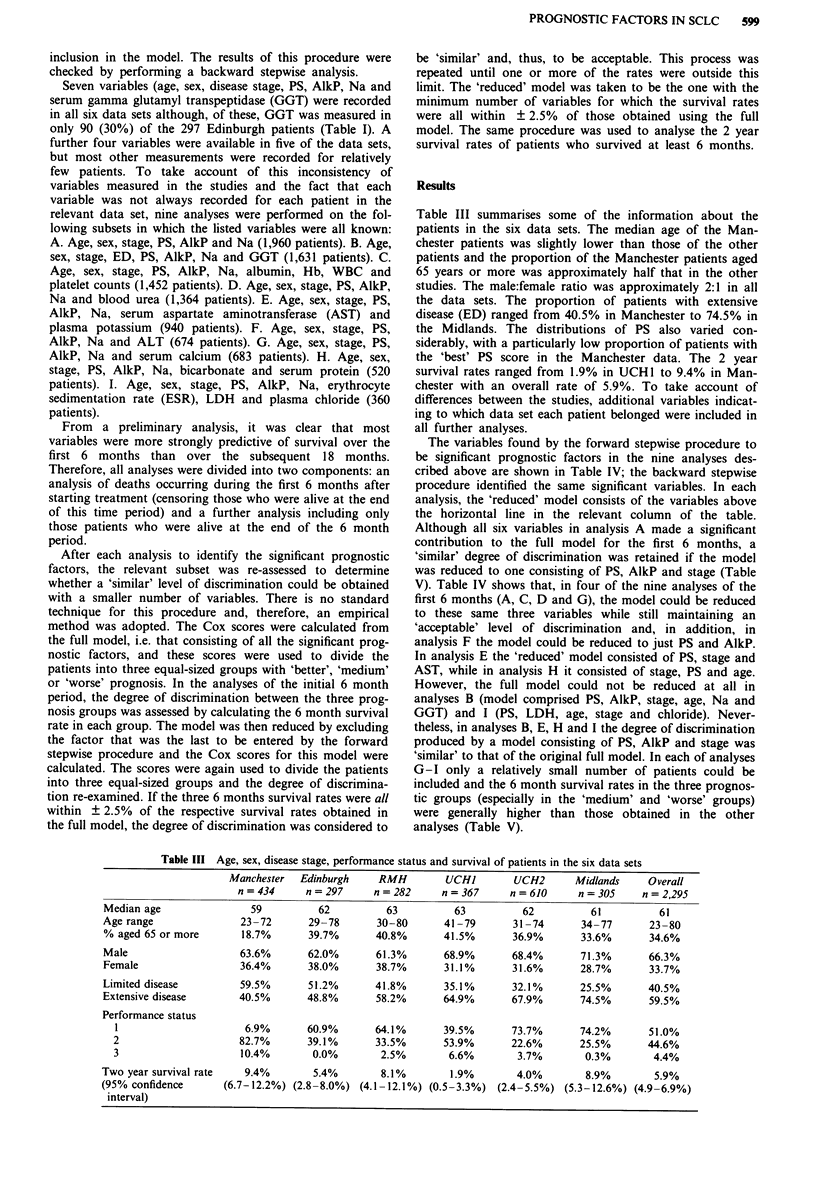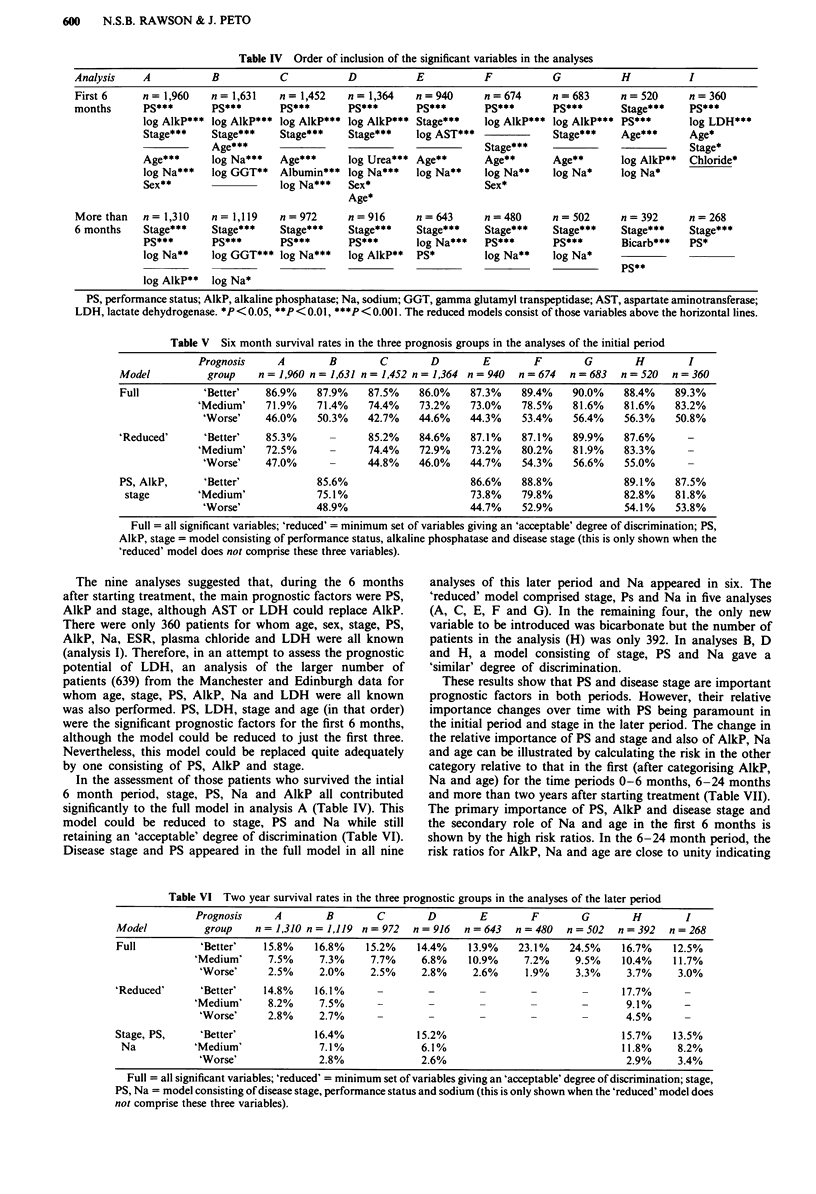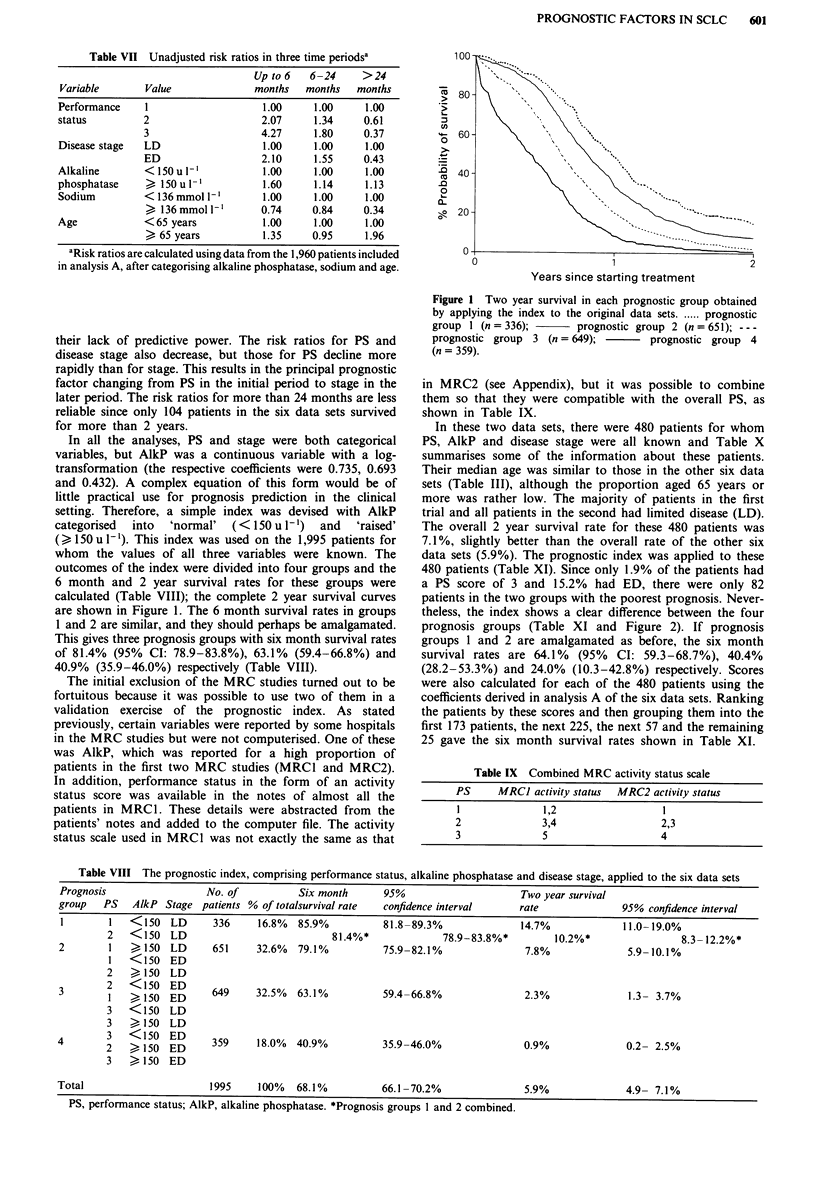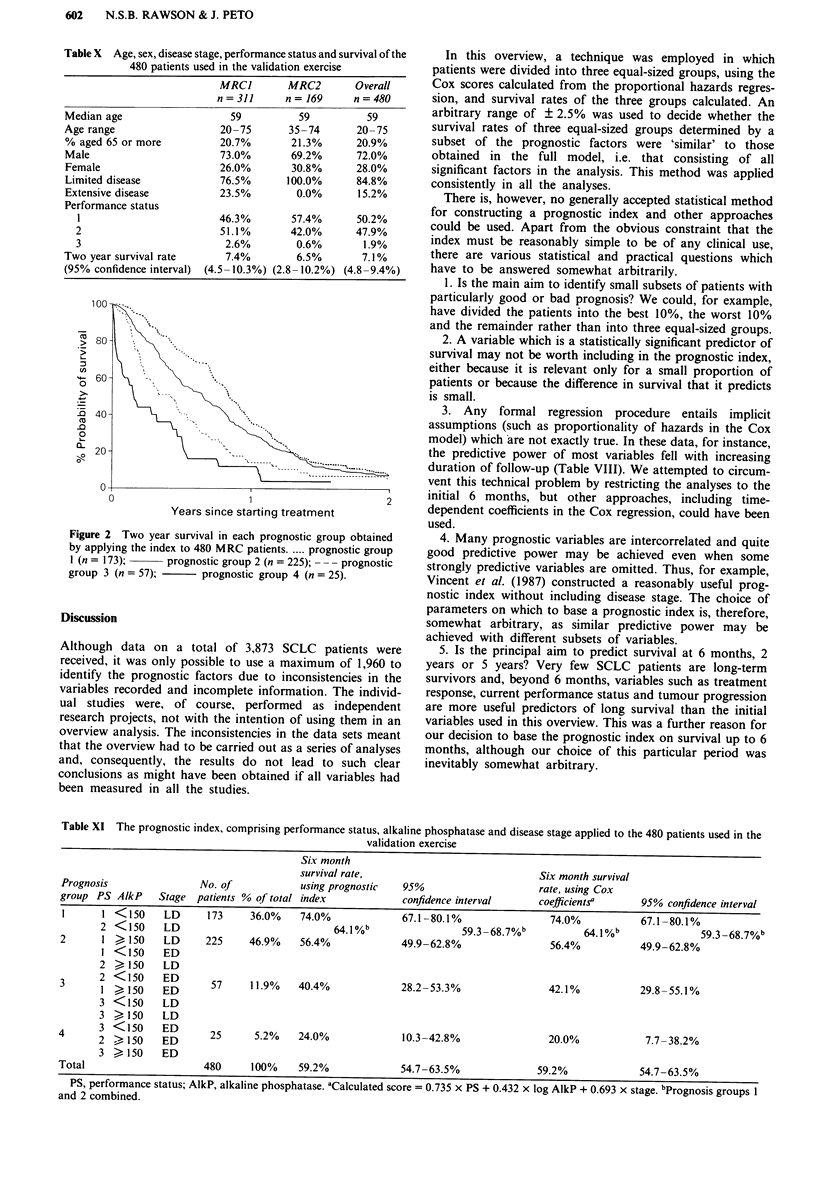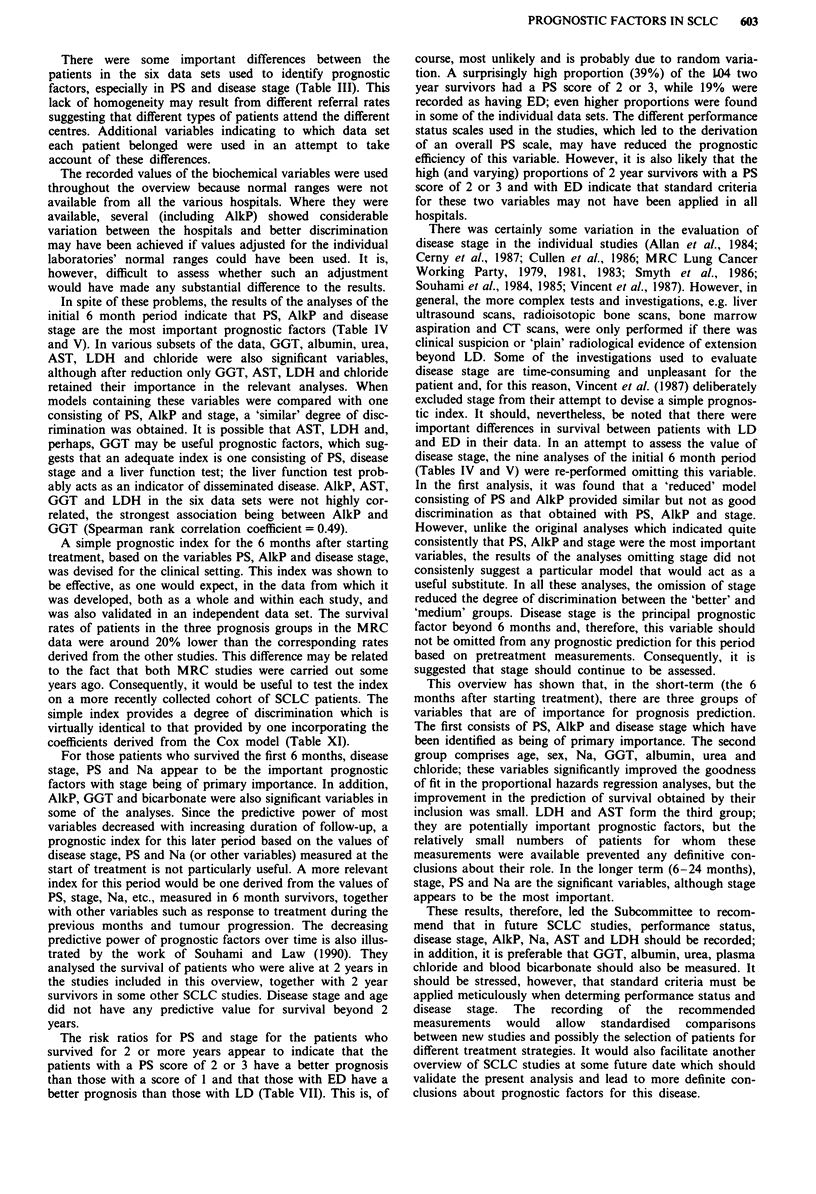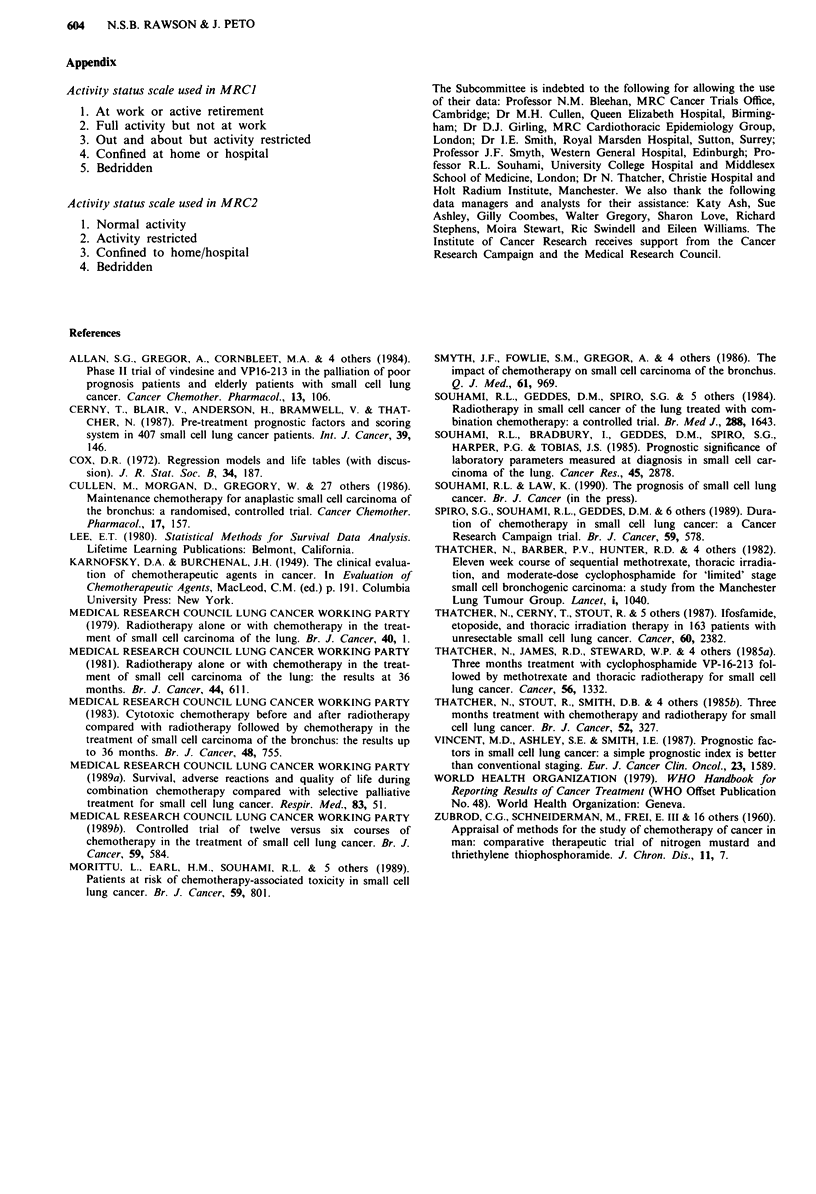# An overview of prognostic factors in small cell lung cancer. A report from the Subcommittee for the Management of Lung Cancer of the United Kingdom Coordinating Committee on Cancer Research.

**DOI:** 10.1038/bjc.1990.133

**Published:** 1990-04

**Authors:** N. S. Rawson, J. Peto

**Affiliations:** Section of Epidemiology, Institute of Cancer Research, Sutton, Surrey, UK.

## Abstract

Several studies of small cell lung cancer (SCLC) treatments have been performed in the United Kingdom. In some, prognostic factor analyses were carried out but the results were not entirely consistent. The Lung Cancer Subcommittee of the United Kingdom Coordinating Committee on Cancer Research (UKCCCR) consequently initiated an overview of these studies with the aim of identifying the important prognostic factors using a large number of patients. Information on almost 4,000 patients was available, but it was necessary to perform analyses on smaller subsets because the variables recorded in individual studies were inconsistent. A number of variables contributed significantly to the prediction of likely survival over the 6 months after starting treatment, but performance status (PS), alkaline phosphatase (AlkP) and disease stage were shown to be the most important; aspartate aminotransferase (AST) and lactate dehydrogenase (LDH) may also be useful. A prognostic index was devised for this initial period and validated using independent data. For patients who survived the first 6 months, the pre-treatment variables important for prognosis in the 6-24 month period were stage, PS and plasma sodium (Na). The Subcommittee recommends that performance status, disease stage, AlkP, Na, AST and LDH should be measured in all future SCLC studies to assist comparisons between studies and possibly the selection of patients for different treatment strategies. The additional recording of five other variables would allow a more definitive overview to be performed at some future date.


*Br. J. Cancer* (1990), **61**, 597–604 © Macmillan Press Ltd., 1990

### An overview of prognostic factors in small cell lung cancer

### A report from the Subcommittee for the Management of Lung Cancer* of the United Kingdom Coordinating Committee on Cancer Research

N.S.B. Rawson† & J. Peto

*Section of Epidemiology, Institute of Cancer Research, Block D, 15 Cotswold Road, Sutton, Surrey SM2 5NG, UK.*

**Summary** Several studies of small cell lung cancer (SCLC) treatments have been performed in the United Kingdom. In some, prognostic factor analyses were carried out but the results were not entirely consistent. The Lung Cancer Subcommittee of the United Kingdom Coordinating Committee on Cancer Research (UKCCCR) consequently initiated an overview of these studies with the aim of identifying the important prognostic factors using a large number of patients. Information on almost 4,000 patients was available, but it was necessary to perform analyses on smaller subsets because the variables recorded in individual studies were inconsistent. A number of variables contributed significantly to the prediction of likely survival over the 6 months after starting treatment, but performance status (PS), alkaline phosphatase (AlkP) and disease stage were shown to be the most important; aspartate aminotransferase (AST) and lactate dehydrogenase (LDH) may also be useful. A prognostic index was devised for this initial period and validated using independent data. For patients who survived the first 6 months, the pre-treatment variables important for prognosis in the 6–24 month period were stage, PS and plasma sodium (Na). The Subcommittee recommends that performance status, disease stage, AlkP, Na, AST and LDH should be measured in all future SCLC studies to assist comparisons between studies and possibly the selection of patients for different treatment strategies. The additional recording of five other variables would allow a more definitive overview to be performed at some future date.

Small cell lung cancer (SCLC) is a disease in which long-term survivors are few, the majority of patients dying within 2 years of diagnosis. Nevertheless, the identification of factors that predict likely survival, especially in the short-term, is of clinical importance since the treatment administered to a patient may depend on the prediction.

A number of studies of SCLC treatments have been carried out in the United Kingdom (Allan *et al.*, 1984; Cullen *et al.*, 1986; MRC Lung Cancer Working Party, 1979, 1981, 1983, 1989*a*, *b*; Smyth *et al.*, 1986; Souhami *et al.*, 1984; Thatcher *et al.*, 1982, 1985*a*, *b*, 1987). In some of these, analyses to determine important prognostic factors were also performed (Cerny *et al.*, 1987; MRC Lung Cancer Working Party, 1981, 1983, 1989*a*, *b*; Souhami *et al.*, 1985; Vincent *et al.*, 1987). Several variables were demonstrated to be of importance in prognosis; these included performance status, disease stage, serum alkaline phosphatase (AlkP), plasma sodium (Na), serum albumin, serum lactate dehydrogenase (LDH), serum alanine aminotransferase (ALT), blood bicarbonate and age. However, the variables recorded and the methods of analysis differed between the studies, and the results were not entirely consistent.

To identify the important prognostic factors using a much larger number of patients than was available in the individual studies, the Subcommittee for the Management of Lung Cancer of the United Kingdom Coordinating Committee on Cancer Research (UKCCCR) initiated an overview of United Kingdom SCLC studies. The participating centres supplied data sets on magnetic media to the Section of Epidemiology at the Institute of Cancer Research for the analysis.

### Patients and methods

Information on a total of 3,873 SCLC patients was received from six centres as follows.

1. Results of four sequential studies, comprising a total of 434 patients treated in two hospitals, were received from the CRC Department of Medical Oncology, Christie Hospital and Holt Radium Institute, Manchester (Thatcher *et al.*, 1982, 1985*a*, *b*, 1987). Since it had been shown previously that outcome was unrelated to treatment in these studies, the results were included as one data set. Up to 61 variables were recorded for each patient. LDH, disease stage, Na, Karnofsky performance status, AlkP and bicarbonate were reported to be the main prognostic factors (Cerny *et al.*, 1987).

2. Data from three non-randomised studies, comprising a total of 297 patients, were received from the ICRF Medical Oncology Unit, Western General Hospital, Edinburgh (Allan *et al.*, 1984; Smyth *et al.*, 1986). There were no differences in outcome between the three treatment regimes and, therefore, the 297 patients were treated as one data set.

3. Data were received for 282 patients referred to the Lung Unit at the Royal Marsden Hospital (RMH), Sutton, Surrey, between 1978 and 1985. The patients were treated according to a series of different chemotherapy protocols which ran consecutively during this period, but outcome was not related to any of the various treatments. Vincent *et al.* (1987) have previously performed a prognostic factor analysis on these data. They had 22 variables available for analysis, but deliberately omitted disease stage from their multivariate analysis. The evaluation of disease stage is often based on time-consuming and elaborate investigations and the aim of these authors was to devise a simple prognostic index excluding this variable. The multivariate analysis showed that albumin, ALT and World Health Organization (WHO) per-

*Members: Professor J.F. Smyth (chairman), Dr N. Thatcher (secretary), Dr D.V. Ash, Professor N.M. Bleehan, Dr. R.L. Carter, Mr P. Goldstraw, Professor S.B. Kaye, Professor J. Peto, Dr I.E. Smith, Dr S. Spiro, Miss D. Watson, Dr J.R. Yarnold, and observers from the Cancer Research Campaign, the Department of Health, the Marie Curie Memorial Foundation, the Medical Research Council, the Scottish Home and Health Department and the Tenovus Institute for Cancer Research.

†Present address: Psychiatric Pharmacoepidemiology Research consortium, Applied Research, University of Saskatchewan, Box 92, University Hospital, Saskatoon, Saskatchewan, S7N 0X0, Canada. Correspondence: J. Peto.

Received 17 July 1989; and in revised form 16 November 1989.

formance status were the most important prognostic factors. However, these variables did not have any predictive value for survival beyond one year, once that had been attained.

4. Results from two multicentre randomised clinical trials, comprising 367 and 610 patients, were received from the Department of Oncology, University College Hospital and Middlesex School of Medicine, London (UCH). There was no difference with regard to survival between the treatment groups in the first trial (Souhami *et al.*, 1984). When the overview analysis was performed, the results from the second were unpublished and the trials were treated as two separate data sets (UCH1 and UCH2) in this work. (The results of the second trial have recently appeared in print (Morittu *et al.*, 1989; Spiro *et al.*, 1989) showing a difference in terms of overall survival between one of the treatment options and the other three. In retrospect, it may have been preferable to include an additional variable to indicate which treatment option was given in this trial). Eleven variables from the first trial have been analysed previously and Karnofsky performance status, AlkP, disease stage, Na and albumin were found to be the main prognostic factors (Souhami *et al.*, 1985). In the second trial, patients who died within three weeks of starting treatment were more likely to have a poor performance status, raised AlkP, raised blood urea and low albumin levels (Morittu *et al.*, 1989).

5. Data on 312 patients included in a multicentre sequential study were received from the Midlands Small Cell Lung Cancer Group, Queen Elizabeth Hospital, Birmingham (Cullen *et al.*, 1986). Ninety-three of the patients were subsequently entered into a randomised trial.

6. Data from four multicentre randomised clinical trials, comprising 330, 193, 706 and 342 patients, were received from the MRC Cardiothoracic Epidemiology Group, Brompton Hospital, London. Details of the first three have been published (MRC Lung Cancer Working Party, 1979, 1981, 1983, 1989*a*, *b*), while the fourth is on-going and the data received comprised those patients entered in the trial by early 1988.

The variables that were available in two or more data sets are shown in Table I. The MRC data included only age, sex and disease stage for all four of its trials, and an activity status score, haemoglobin (Hb), white blood cell (WBC) and platelet counts in three of the four (Table I); other measurements were recorded by some of the participating hospitals, but they were not computerised. Since the aim of the overview was to assess as many potential prognostic factors as possible, it was decided to exclude the MRC studies. This decision was fortuitous because it was subsequently possible to use informaton on patients in two of the MRC studies in a validation exercise. After excluding the MRC studies, there remained a total of 2,302 patients from the other six data sets. Survival data for seven patients were incomplete and the prognostic variables that were measured in each of the six data sets were not recorded for every patient. The maximum number of patients included in any prognostic analysis was 1,960.

Performance status was not measured in the same way in each of the six data sets. In Manchester and UCH1, the Karnofsky scale (Karnofsky & Burchenal, 1949) was used, while in the other four either the Eastern Cooperative Oncology Group (ECOG) scale (Zubrod *et al.*, 1960) or the WHO scale (World Health Organization, 1979) was used. Apart from minor differences in the wording of the categories, the ECOG and WHO scales are the same, but the Karnofsky scale is different. Therefore, the overall assessment of performance status (PS) shown in Table II was devised and used throughout the analysis.

The principal method of analysis used in this overview was proportional hazards regression, which is a multiple regression technique for investigating the relationship between survival time and possible explanatory (prognostic) variables (Cox, 1972). Using this method, regression coefficients are estimated and a prognostic index or 'Cox score', which is the logarithm of the individual's predicted relative death rate, can be calculated for each patient. A forward stepwise procedure, in which independent variables are added to the regression equation one at a time, was used to identify the significant prognostic factors. At each step, the independent variable which gave the largest increase in log-likelihood was entered into the model if the likelihood ratio test showed that the variable significantly improved the goodness of fit (Lee, 1980). A significance level of 5% was set as the limit for

**Table II** Overall assessment of performance status

| *PS* | *ECOG* | *WHO* | *Karnofsky* |
|---|---|---|---|
| 1 | 0,1 | 0,1 | 80–100 |
| 2 | 2,3 | 2,3 | 50–70 |
| 3 | 4 | 4 | 10–40 |

**Table I** Variables recorded in the 10 data sets

| *Variable* | *Manchester n = 434* | *Edinburgh n = 297* | *RMH n = 282* | *UCH1 n = 367* | *UCH2 n = 610* | *Midlands n = 312* | *MRC1 n = 330* | *MRC2 n = 193* | *MRC3 n = 706* | *MRC4 n = 342* |
|---|---|---|---|---|---|---|---|---|---|---|
| Age | Yes | Yes | Yes | Yes | Yes | Yes | Yes | Yes | Yes | Yes |
| Sex | Yes | Yes | Yes | Yes | Yes | Yes | Yes | Yes | Yes | Yes |
| Disease stage | Yes | Yes | Yes | Yes | Yes | Yes | Yes | Yes | Yes | Yes |
| Performance status (PS) | Yes | Yes | Yes | Yes | Yes | Yes | No | Yes | Yes | Yes |
| Serum alkaline phosphatase (AlkP) | Yes | Yes | Yes | Yes | Yes | Yes | No | No | No | No |
| Plasma sodium (Na) | Yes | Yes | Yes | Yes | Yes | Yes | No | No | No | No |
| Gamma glutamyl transpeptidase (GGT) | Yes | Yes[a] | Yes | Yes | Yes | Yes | No | No | No | No |
| Serum albumin | Yes | Yes | Yes | Yes | Yes | No | No | No | No | No |
| Haemoglobin (Hb) | Yes | Yes | Yes | Yes | Yes | No | No | Yes | Yes | Yes |
| White blood cells (WBC) | Yes | Yes | Yes | Yes | Yes | No | No | Yes | Yes | Yes |
| Platelets | Yes | Yes | Yes | Yes | Yes | No | No | Yes | Yes | Yes |
| Blood urea | Yes | No | No | Yes | Yes | Yes | No | No | No | No |
| Aspartate aminotransferase (AST) | Yes | Yes | No | Yes | No | No | No | No | No | No |
| Plasma potassium | Yes | Yes | No | Yes | No | No | No | No | No | No |
| Alanine aminotransferase (ALT) | Yes | No | Yes | Yes | No | No | No | No | No | No |
| Serum calcium | Yes | No | Yes | No | No | No | No | No | No | No |
| Blood bicarbonate | Yes | No | No | Yes | No | No | No | No | No | No |
| Serum protein | Yes | No | No | Yes | No | No | No | No | No | No |
| Erythrocyte sedimentation rate (ESR) | Yes | Yes | No | No | No | No | No | No | No | No |
| Plasma chloride | Yes | Yes | No | No | No | No | No | No | No | No |
| Serum lactate dehydrogenase (LDH) | Yes | Yes | No | No | No | No | No | No | No | No |

[a]Values for only 90 of these patients were recorded inclusion in the model. The results of this procedure were checked by performing a backward stepwise analysis.

Seven variables (age, sex, disease stage, PS, AlkP, Na and serum gamma glutamyl transpeptidase (GGT) were recorded in all six data sets although, of these, GGT was measured in only 90 (30%) of the 297 Edinburgh patients (Table I). A further four variables were available in five of the data sets, but most other measurements were recorded for relatively few patients. To take account of this inconsistency of variables measured in the studies and the fact that each variable was not always recorded for each patient in the relevant data set, nine analyses were performed on the following subsets in which the listed variables were all known: A. Age, sex, stage, PS, AlkP and Na (1,960 patients). B. Age, sex, stage, ED, PS, AlkP, Na and GGT (1,631 patients). C. Age, sex, stage, PS, AlkP, Na, albumin, Hb, WBC and platelet counts (1,452 patients). D. Age, sex, stage, PS, AlkP, Na and blood urea (1,364 patients). E. Age, sex, stage, PS, AlkP, Na, serum aspartate aminotransferase (AST) and plasma potassium (940 patients). F. Age, sex, stage, PS, AlkP, Na and ALT (674 patients). G. Age, sex, stage, PS, AlkP, Na and serum calcium (683 patients). H. Age, sex, stage, PS, AlkP, Na, bicarbonate and serum protein (520 patients). I. Age, sex, stage, PS, AlkP, Na, erythrocyte sedimentation rate (ESR), LDH and plasma chloride (360 patients).

From a preliminary analysis, it was clear that most variables were more strongly predictive of survival over the first 6 months than over the subsequent 18 months. Therefore, all analyses were divided into two components: an analysis of deaths occurring during the first 6 months after starting treatment (censoring those who were alive at the end of this time period) and a further analysis including only those patients who were alive at the end of the 6 month period.

After each analysis to identify the significant prognostic factors, the relevant subset was re-assessed to determine whether a 'similar' level of discrimination could be obtained with a smaller number of variables. There is no standard technique for this procedure and, therefore, an empirical method was adopted. The Cox scores were calculated from the full model, i.e. that consisting of all the significant prognostic factors, and these scores were used to divide the patients into three equal-sized groups with 'better', 'medium' or 'worse' prognosis. In the analyses of the initial 6 month period, the degree of discrimination between the three prognosis groups was assessed by calculating the 6 month survival rate in each group. The model was then reduced by excluding the factor that was the last to be entered by the forward stepwise procedure and the Cox scores for this model were calculated. The scores were again used to divide the patients into three equal-sized groups and the degree of discrimination re-examined. If the three 6 months survival rates were *all* within ± 2.5% of the respective survival rates obtained in the full model, the degree of discrimination was considered to be 'similar' and, thus, to be acceptable. This process was repeated until one or more of the rates were outside this limit. The 'reduced' model was taken to be the one with the minimum number of variables for which the survival rates were all within ± 2.5% of those obtained using the full model. The same procedure was used to analyse the 2 year survival rates of patients who survived at least 6 months.

### Results

Table III summarises some of the information about the patients in the six data sets. The median age of the Manchester patients was slightly lower than those of the other patients and the proportion of the Manchester patients aged 65 years or more was approximately half that in the other studies. The male:female ratio was approximately 2:1 in all the data sets. The proportion of patients with extensive disease (ED) ranged from 40.5% in Manchester to 74.5% in the Midlands. The distributions of PS also varied considerably, with a particularly low proportion of patients with the 'best' PS score in the Manchester data. The 2 year survival rates ranged from 1.9% in UCH1 to 9.4% in Manchester with an overall rate of 5.9%. To take account of differences between the studies, additional variables indicating to which data set each patient belonged were included in all further analyses.

The variables found by the forward stepwise procedure to be significant prognostic factors in the nine analyses described above are shown in Table IV; the backward stepwise procedure identified the same significant variables. In each analysis, the 'reduced' model consists of the variables above the horizontal line in the relevant column of the table. Although all six variables in analysis A made a significant contribution to the full model for the first 6 months, a 'similar' degree of discrimination was retained if the model was reduced to one consisting of PS, AlkP and stage (Table V). Table IV shows that, in four of the nine analyses of the first 6 months (A, C, D and G), the model could be reduced to these same three variables while still maintaining an 'acceptable' level of discrimination and, in addition, in analysis F the model could be reduced to just PS and AlkP. In analysis E the 'reduced' model consisted of PS, stage and AST, while in analysis H it consisted of stage, PS and age. However, the full model could not be reduced at all in analyses B (model comprised PS, AlkP, stage, age, Na and GGT) and I (PS, LDH, age, stage and chloride). Nevertheless, in analyses B, E, H and I the degree of discrimination produced by a model consisting of PS, AlkP and stage was 'similar' to that of the original full model. In each of analyses G–I only a relatively small number of patients could be included and the 6 month survival rates in the three prognostic groups (especially in the 'medium' and 'worse' groups) were generally higher than those obtained in the other analyses (Table V).

**Table III** Age, sex, disease stage, performance status and survival of patients in the six data sets

| | *Manchester* $n = 434$ | *Edinburgh* $n = 297$ | *RMH* $n = 282$ | *UCH1* $n = 367$ | *UCH2* $n = 610$ | *Midlands* $n = 305$ | *Overall* $n = 2,295$ |
|---|---|---|---|---|---|---|---|
| Median age | 59 | 62 | 63 | 63 | 62 | 61 | 61 |
| Age range | 23–72 | 29–78 | 30–80 | 41–79 | 31–74 | 34–77 | 23–80 |
| % aged 65 or more | 18.7% | 39.7% | 40.8% | 41.5% | 36.9% | 33.6% | 34.6% |
| Male | 63.6% | 62.0% | 61.3% | 68.9% | 68.4% | 71.3% | 66.3% |
| Female | 36.4% | 38.0% | 38.7% | 31.1% | 31.6% | 28.7% | 33.7% |
| Limited disease | 59.5% | 51.2% | 41.8% | 35.1% | 32.1% | 25.5% | 40.5% |
| Extensive disease | 40.5% | 48.8% | 58.2% | 64.9% | 67.9% | 74.5% | 59.5% |
| Performance status | | | | | | | |
| 1 | 6.9% | 60.9% | 64.1% | 39.5% | 73.7% | 74.2% | 51.0% |
| 2 | 82.7% | 39.1% | 33.5% | 53.9% | 22.6% | 25.5% | 44.6% |
| 3 | 10.4% | 0.0% | 2.5% | 6.6% | 3.7% | 0.3% | 4.4% |
| Two year survival rate | 9.4% | 5.4% | 8.1% | 1.9% | 4.0% | 8.9% | 5.9% |
| (95% confidence interval) | (6.7–12.2%) | (2.8–8.0%) | (4.1–12.1%) | (0.5–3.3%) | (2.4–5.5%) | (5.3–12.6%) | (4.9–6.9%) |

**Table IV** Order of inclusion of the significant variables in the analyses

| *Analysis* | *A* | *B* | *C* | *D* | *E* | *F* | *G* | *H* | *I* |
|---|---|---|---|---|---|---|---|---|---|
| First 6 months | *n* = 1,960 | *n* = 1,631 | *n* = 1,452 | *n* = 1,364 | *n* = 940 | *n* = 674 | *n* = 683 | *n* = 520 | *n* = 360 |
| | PS*** | PS*** | PS*** | PS*** | PS*** | PS*** | PS*** | Stage*** | PS*** |
| | log AlkP*** | log AlkP*** | log AlkP*** | log AlkP*** | Stage*** | log AlkP*** | log AlkP*** | PS*** | log LDH*** |
| | Stage*** | Stage*** | Stage*** | Stage*** | log AST*** | ——— | Stage*** | Age*** | Age* |
| | ——— | Age*** | ——— | ——— | ——— | Stage*** | ——— | ——— | Stage* |
| | Age*** | log Na*** | Age*** | log Urea*** | Age** | Age** | Age** | log AlkP** | Chloride* |
| | log Na*** | log GGT** | Albumin*** | log Na*** | log Na** | log Na** | log Na* | log Na* | ——— |
| | Sex** | ——— | log Na*** | Sex* | | Sex* | | | |
| | | | | Age* | | | | | |
| More than 6 months | *n* = 1,310 | *n* = 1,119 | *n* = 972 | *n* = 916 | *n* = 643 | *n* = 480 | *n* = 502 | *n* = 392 | *n* = 268 |
| | Stage*** | Stage*** | Stage*** | Stage*** | Stage*** | Stage*** | Stage*** | Stage*** | Stage*** |
| | PS*** | PS*** | PS*** | PS*** | log Na*** | PS*** | PS*** | Bicarb*** | PS* |
| | log Na** | log GGT*** | log Na*** | log AlkP** | PS* | log Na** | log Na* | ——— | ——— |
| | ——— | ——— | ——— | ——— | ——— | ——— | ——— | PS** | |
| | log AlkP** | log Na* | | | | | | | |

PS, performance status; AlkP, alkaline phosphatase; Na, sodium; GGT, gamma glutamyl transpeptidase; AST, aspartate aminotransferase; LDH, lactate dehydrogenase. $^{*}P<0.05$, $^{**}P<0.01$, $^{***}P<0.001$. The reduced models consist of those variables above the horizontal lines.

**Table V** Six month survival rates in the three prognosis groups in the analyses of the initial period

| *Model* | *Prognosis group* | *A* *n = 1,960* | *B* *n = 1,631* | *C* *n = 1,452* | *D* *n = 1,364* | *E* *n = 940* | *F* *n = 674* | *G* *n = 683* | *H* *n = 520* | *I* *n = 360* |
|---|---|---|---|---|---|---|---|---|---|---|
| Full | 'Better' | 86.9% | 87.9% | 87.5% | 86.0% | 87.3% | 89.4% | 90.0% | 88.4% | 89.3% |
| | 'Medium' | 71.9% | 71.4% | 74.4% | 73.2% | 73.0% | 78.5% | 81.6% | 81.6% | 83.2% |
| | 'Worse' | 46.0% | 50.3% | 42.7% | 44.6% | 44.3% | 53.4% | 56.4% | 56.3% | 50.8% |
| 'Reduced' | 'Better' | 85.3% | – | 85.2% | 84.6% | 87.1% | 87.1% | 89.9% | 87.6% | – |
| | 'Medium' | 72.5% | – | 74.4% | 72.9% | 73.2% | 80.2% | 81.9% | 83.3% | – |
| | 'Worse' | 47.0% | – | 44.8% | 46.0% | 44.7% | 54.3% | 56.6% | 55.0% | – |
| PS, AlkP, stage | 'Better' | | 85.6% | | | 86.6% | 88.8% | | 89.1% | 87.5% |
| | 'Medium' | | 75.1% | | | 73.8% | 79.8% | | 82.8% | 81.8% |
| | 'Worse' | | 48.9% | | | 44.7% | 52.9% | | 54.1% | 53.8% |

Full = all significant variables; 'reduced' = minimum set of variables giving an 'acceptable' degree of discrimination; PS, AlkP, stage = model consisting of performance status, alkaline phosphatase and disease stage (this is only shown when the 'reduced' model does *not* comprise these three variables).

The nine analyses suggested that, during the 6 months after starting treatment, the main prognostic factors were PS, AlkP and stage, although AST or LDH could replace AlkP. There were only 360 patients for whom age, sex, stage, PS, AlkP, Na, ESR, plasma chloride and LDH were all known (analysis I). Therefore, in an attempt to assess the prognostic potential of LDH, an analysis of the larger number of patients (639) from the Manchester and Edinburgh data for whom age, stage, PS, AlkP, Na and LDH were all known was also performed. PS, LDH, stage and age (in that order) were the significant prognostic factors for the first 6 months, although the model could be reduced to just the first three. Nevertheless, this model could be replaced quite adequately by one consisting of PS, AlkP and stage.

In the assessment of those patients who survived the intial 6 month period, stage, PS, Na and AlkP all contributed significantly to the full model in analysis A (Table IV). This model could be reduced to stage, PS and Na while still retaining an 'acceptable' degree of discrimination (Table VI). Disease stage and PS appeared in the full model in all nine analyses of this later period and Na appeared in six. The 'reduced' model comprised stage, Ps and Na in five analyses (A, C, E, F and G). In the remaining four, the only new variable to be introduced was bicarbonate but the number of patients in the analysis (H) was only 392. In analyses B, D and H, a model consisting of stage, PS and Na gave a 'similar' degree of discrimination.

These results show that PS and disease stage are important prognostic factors in both periods. However, their relative importance changes over time with PS being paramount in the initial period and stage in the later period. The change in the relative importance of PS and stage and also of AlkP, Na and age can be illustrated by calculating the risk in the other category relative to that in the first (after categorising AlkP, Na and age) for the time periods 0–6 months, 6–24 months and more than two years after starting treatment (Table VII). The primary importance of PS, AlkP and disease stage and the secondary role of Na and age in the first 6 months is shown by the high risk ratios. In the 6–24 month period, the risk ratios for AlkP, Na and age are close to unity indicating

**Table VI** Two year survival rates in the three prognostic groups in the analyses of the later period

| *Model* | *Prognosis group* | *A* *n = 1,310* | *B* *n = 1,119* | *C* *n = 972* | *D* *n = 916* | *E* *n = 643* | *F* *n = 480* | *G* *n = 502* | *H* *n = 392* | *I* *n = 268* |
|---|---|---|---|---|---|---|---|---|---|---|
| Full | 'Better' | 15.8% | 16.8% | 15.2% | 14.4% | 13.9% | 23.1% | 24.5% | 16.7% | 12.5% |
| | 'Medium' | 7.5% | 7.3% | 7.7% | 6.8% | 10.9% | 7.2% | 9.5% | 10.4% | 11.7% |
| | 'Worse' | 2.5% | 2.0% | 2.5% | 2.8% | 2.6% | 1.9% | 3.3% | 3.7% | 3.0% |
| 'Reduced' | 'Better' | 14.8% | 16.1% | – | – | – | – | – | 17.7% | – |
| | 'Medium' | 8.2% | 7.5% | – | – | – | – | – | 9.1% | – |
| | 'Worse' | 2.8% | 2.7% | – | – | – | – | – | 4.5% | – |
| Stage, PS, Na | 'Better' | | 16.4% | | 15.2% | | | | 15.7% | 13.5% |
| | 'Medium' | | 7.1% | | 6.1% | | | | 11.8% | 8.2% |
| | 'Worse' | | 2.8% | | 2.6% | | | | 2.9% | 3.4% |

Full = all significant variables; 'reduced' = minimum set of variables giving an 'acceptable' degree of discrimination; stage, PS, Na = model consisting of disease stage, performance status and sodium (this is only shown when the 'reduced' model does *not* comprise these three variables).

**Table VII** Unadjusted risk ratios in three time periods[a]

| *Variable* | *Value* | *Up to 6 months* | *6–24 months* | *>24 months* |
|---|---|---|---|---|
| Performance status | 1 | 1.00 | 1.00 | 1.00 |
| | 2 | 2.07 | 1.34 | 0.61 |
| | 3 | 4.27 | 1.80 | 0.37 |
| Disease stage | LD | 1.00 | 1.00 | 1.00 |
| | ED | 2.10 | 1.55 | 0.43 |
| Alkaline phosphatase | $< 150\ u\ l^{-1}$ | 1.00 | 1.00 | 1.00 |
| | $\geqslant 150\ u\ l^{-1}$ | 1.60 | 1.14 | 1.13 |
| Sodium | $< 136\ mmol\ l^{-1}$ | 1.00 | 1.00 | 1.00 |
| | $\geqslant 136\ mmol\ l^{-1}$ | 0.74 | 0.84 | 0.34 |
| Age | <65 years | 1.00 | 1.00 | 1.00 |
| | ⩾ 65 years | 1.35 | 0.95 | 1.96 |

[a]Risk ratios are calculated using data from the 1,960 patients included in analysis A, after categorising alkaline phosphatase, sodium and age.

**Figure 1** Two year survival in each prognostic group obtained by applying the index to the original data sets. ..... prognostic group 1 ($n = 336$); ——— prognostic group 2 ($n = 651$); - - - prognostic group 3 ($n = 649$); ——— prognostic group 4 ($n = 359$).

their lack of predictive power. The risk ratios for PS and disease stage also decrease, but those for PS decline more rapidly than for stage. This results in the principal prognostic factor changing from PS in the initial period to stage in the later period. The risk ratios for more than 24 months are less reliable since only 104 patients in the six data sets survived for more than 2 years.

In all the analyses, PS and stage were both categorical variables, but AlkP was a continuous variable with a log-transformation (the respective coefficients were 0.735, 0.693 and 0.432). A complex equation of this form would be of little practical use for prognosis prediction in the clinical setting. Therefore, a simple index was devised with AlkP categorised into 'normal' ($<150\ u\ l^{-1}$) and 'raised' ($\geqslant 150\ u\ l^{-1}$). This index was used on the 1,995 patients for whom the values of all three variables were known. The outcomes of the index were divided into four groups and the 6 month and 2 year survival rates for these groups were calculated (Table VIII); the complete 2 year survival curves are shown in Figure 1. The 6 month survival rates in groups 1 and 2 are similar, and they should perhaps be amalgamated. This gives three prognosis groups with six month survival rates of 81.4% (95% CI: 78.9–83.8%), 63.1% (59.4–66.8%) and 40.9% (35.9–46.0%) respectively (Table VIII).

The initial exclusion of the MRC studies turned out to be fortuitous because it was possible to use two of them in a validation exercise of the prognostic index. As stated previously, certain variables were reported by some hospitals in the MRC studies but were not computerised. One of these was AlkP, which was reported for a high proportion of patients in the first two MRC studies (MRC1 and MRC2). In addition, performance status in the form of an activity status score was available in the notes of almost all the patients in MRC1. These details were abstracted from the patients' notes and added to the computer file. The activity status scale used in MRC1 was not exactly the same as that in MRC2 (see Appendix), but it was possible to combine them so that they were compatible with the overall PS, as shown in Table IX.

In these two data sets, there were 480 patients for whom PS, AlkP and disease stage were all known and Table X summarises some of the information about these patients. Their median age was similar to those in the other six data sets (Table III), although the proportion aged 65 years or more was rather low. The majority of patients in the first trial and all patients in the second had limited disease (LD). The overall 2 year survival rate for these 480 patients was 7.1%, slightly better than the overall rate of the other six data sets (5.9%). The prognostic index was applied to these 480 patients (Table XI). Since only 1.9% of the patients had a PS score of 3 and 15.2% had ED, there were only 82 patients in the two groups with the poorest prognosis. Nevertheless, the index shows a clear difference between the four prognosis groups (Table XI and Figure 2). If prognosis groups 1 and 2 are amalgamated as before, the six month survival rates are 64.1% (95% CI: 59.3–68.7%), 40.4% (28.2–53.3%) and 24.0% (10.3–42.8%) respectively. Scores were also calculated for each of the 480 patients using the coefficients derived in analysis A of the six data sets. Ranking the patients by these scores and then grouping them into the first 173 patients, the next 225, the next 57 and the remaining 25 gave the six month survival rates shown in Table XI.

**Table IX** Combined MRC activity status scale

| *PS* | *MRC1 activity status* | *MRC2 activity status* |
|---|---|---|
| 1 | 1,2 | 1 |
| 2 | 3,4 | 2,3 |
| 3 | 5 | 4 |

**Table VIII** The prognostic index, comprising performance status, alkaline phosphatase and disease stage, applied to the six data sets

| *Prognosis group* | *PS* | *AlkP* | *Stage* | *No. of patients* | *% of total* | *Six month survival rate* | *95% confidence interval* | *Two year survival rate* | *95% confidence interval* |
|---|---|---|---|---|---|---|---|---|---|
| 1 | 1 | <150 | LD | 336 | 16.8% | 85.9% | 81.8–89.3% | 14.7% | 11.0–19.0% |
| | 2 | <150 | LD | | | 81.4%* | 78.9–83.8%* | 10.2%* | 8.3–12.2%* |
| 2 | 1 | ⩾150 | LD | 651 | 32.6% | 79.1% | 75.9–82.1% | 7.8% | 5.9–10.1% |
| | 1 | <150 | ED | | | | | | |
| | 2 | ⩾150 | LD | | | | | | |
| 3 | 2 | <150 | ED | 649 | 32.5% | 63.1% | 59.4–66.8% | 2.3% | 1.3– 3.7% |
| | 1 | ⩾150 | ED | | | | | | |
| | 3 | <150 | LD | | | | | | |
| | 3 | ⩾150 | LD | | | | | | |
| 4 | 3 | <150 | ED | 359 | 18.0% | 40.9% | 35.9–46.0% | 0.9% | 0.2– 2.5% |
| | 2 | ⩾150 | ED | | | | | | |
| | 3 | ⩾150 | ED | | | | | | |
| Total | | | | 1995 | 100% | 68.1% | 66.1–70.2% | 5.9% | 4.9– 7.1% |

PS, performance status; AlkP, alkaline phosphatase. *Prognosis groups 1 and 2 combined.

**Table X** Age, sex, disease stage, performance status and survival of the 480 patients used in the validation exercise

| | *MRC1* *n = 311* | *MRC2* *n = 169* | *Overall* *n = 480* |
|---|---|---|---|
| Median age | 59 | 59 | 59 |
| Age range | 20–75 | 35–74 | 20–75 |
| % aged 65 or more | 20.7% | 21.3% | 20.9% |
| Male | 73.0% | 69.2% | 72.0% |
| Female | 26.0% | 30.8% | 28.0% |
| Limited disease | 76.5% | 100.0% | 84.8% |
| Extensive disease | 23.5% | 0.0% | 15.2% |
| Performance status | | | |
| 1 | 46.3% | 57.4% | 50.2% |
| 2 | 51.1% | 42.0% | 47.9% |
| 3 | 2.6% | 0.6% | 1.9% |
| Two year survival rate | 7.4% | 6.5% | 7.1% |
| (95% confidence interval) | (4.5–10.3%) | (2.8–10.2%) | (4.8–9.4%) |

**Figure 2** Two year survival in each prognostic group obtained by applying the index to 480 MRC patients. .... prognostic group 1 ($n = 173$); ——— prognostic group 2 ($n = 225$); - - - prognostic group 3 ($n = 57$); ——— prognostic group 4 ($n = 25$).

### Discussion

Although data on a total of 3,873 SCLC patients were received, it was only possible to use a maximum of 1,960 to identify the prognostic factors due to inconsistencies in the variables recorded and incomplete information. The individual studies were, of course, performed as independent research projects, not with the intention of using them in an overview analysis. The inconsistencies in the data sets meant that the overview had to be carried out as a series of analyses and, consequently, the results do not lead to such clear conclusions as might have been obtained if all variables had been measured in all the studies.

In this overview, a technique was employed in which patients were divided into three equal-sized groups, using the Cox scores calculated from the proportional hazards regression, and survival rates of the three groups calculated. An arbitrary range of ± 2.5% was used to decide whether the survival rates of three equal-sized groups determined by a subset of the prognostic factors were 'similar' to those obtained in the full model, i.e. that consisting of all significant factors in the analysis. This method was applied consistently in all the analyses.

There is, however, no generally accepted statistical method for constructing a prognostic index and other approaches could be used. Apart from the obvious constraint that the index must be reasonably simple to be of any clinical use, there are various statistical and practical questions which have to be answered somewhat arbitrarily.

1. Is the main aim to identify small subsets of patients with particularly good or bad prognosis? We could, for example, have divided the patients into the best 10%, the worst 10% and the remainder rather than into three equal-sized groups.

2. A variable which is a statistically significant predictor of survival may not be worth including in the prognostic index, either because it is relevant only for a small proportion of patients or because the difference in survival that it predicts is small.

3. Any formal regression procedure entails implicit assumptions (such as proportionality of hazards in the Cox model) which are not exactly true. In these data, for instance, the predictive power of most variables fell with increasing duration of follow-up (Table VIII). We attempted to circumvent this technical problem by restricting the analyses to the initial 6 months, but other approaches, including time-dependent coefficients in the Cox regression, could have been used.

4. Many prognostic variables are intercorrelated and quite good predictive power may be achieved even when some strongly predictive variables are omitted. Thus, for example, Vincent *et al.* (1987) constructed a reasonably useful prognostic index without including disease stage. The choice of parameters on which to base a prognostic index is, therefore, somewhat arbitrary, as similar predictive power may be achieved with different subsets of variables.

5. Is the principal aim to predict survival at 6 months, 2 years or 5 years? Very few SCLC patients are long-term survivors and, beyond 6 months, variables such as treatment response, current performance status and tumour progression are more useful predictors of long survival than the initial variables used in this overview. This was a further reason for our decision to base the prognostic index on survival up to 6 months, although our choice of this particular period was inevitably somewhat arbitrary.

**Table XI** The prognostic index, comprising performance status, alkaline phosphatase and disease stage applied to the 480 patients used in the validation exercise

| *Prognosis group* | *PS* | *AlkP* | *Stage* | *No. of patients* | *% of total* | *Six month survival rate, using prognostic index* | *95% confidence interval* | *Six month survival rate, using Cox coefficients*[a] | *95% confidence interval* |
|---|---|---|---|---|---|---|---|---|---|
| 1 | 1 | <150 | LD | 173 | 36.0% | 74.0% | 67.1–80.1% | 74.0% | 67.1–80.1% |
| | 2 | <150 | LD | | | 64.1%[b] | 59.3–68.7%[b] | 64.1%[b] | 59.3–68.7%[b] |
| 2 | 1 | ≥150 | LD | 225 | 46.9% | 56.4% | 49.9–62.8% | 56.4% | 49.9–62.8% |
| | 1 | <150 | ED | | | | | | |
| | 2 | ≥150 | LD | | | | | | |
| 3 | 2 | <150 | ED | 57 | 11.9% | 40.4% | 28.2–53.3% | 42.1% | 29.8–55.1% |
| | 1 | ≥150 | ED | | | | | | |
| | 3 | <150 | LD | | | | | | |
| | 3 | ≥150 | LD | | | | | | |
| 4 | 3 | <150 | ED | 25 | 5.2% | 24.0% | 10.3–42.8% | 20.0% | 7.7–38.2% |
| | 2 | ≥150 | ED | | | | | | |
| | 3 | ≥150 | ED | | | | | | |
| Total | | | | 480 | 100% | 59.2% | 54.7–63.5% | 59.2% | 54.7–63.5% |

PS, performance status; AlkP, alkaline phosphatase. [a]Calculated score = 0.735 × PS + 0.432 × log AlkP + 0.693 × stage. [b]Prognosis groups 1 and 2 combined.

There were some important differences between the patients in the six data sets used to identify prognostic factors, especially in PS and disease stage (Table III). This lack of homogeneity may result from different referral rates suggesting that different types of patients attend the different centres. Additional variables indicating to which data set each patient belonged were used in an attempt to take account of these differences.

The recorded values of the biochemical variables were used throughout the overview because normal ranges were not available from all the various hospitals. Where they were available, several (including AlkP) showed considerable variation between the hospitals and better discrimination may have been achieved if values adjusted for the individual laboratories' normal ranges could have been used. It is, however, difficult to assess whether such an adjustment would have made any substantial difference to the results.

In spite of these problems, the results of the analyses of the initial 6 month period indicate that PS, AlkP and disease stage are the most important prognostic factors (Table IV and V). In various subsets of the data, GGT, albumin, urea, AST, LDH and chloride were also significant variables, although after reduction only GGT, AST, LDH and chloride retained their importance in the relevant analyses. When models containing these variables were compared with one consisting of PS, AlkP and stage, a 'similar' degree of discrimination was obtained. It is possible that AST, LDH and, perhaps, GGT may be useful prognostic factors, which suggests that an adequate index is one consisting of PS, disease stage and a liver function test; the liver function test probably acts as an indicator of disseminated disease. AlkP, AST, GGT and LDH in the six data sets were not highly correlated, the strongest association being between AlkP and GGT (Spearman rank correlation coefficient = 0.49).

A simple prognostic index for the 6 months after starting treatment, based on the variables PS, AlkP and disease stage, was devised for the clinical setting. This index was shown to be effective, as one would expect, in the data from which it was developed, both as a whole and within each study, and was also validated in an independent data set. The survival rates of patients in the three prognosis groups in the MRC data were around 20% lower than the corresponding rates derived from the other studies. This difference may be related to the fact that both MRC studies were carried out some years ago. Consequently, it would be useful to test the index on a more recently collected cohort of SCLC patients. The simple index provides a degree of discrimination which is virtually identical to that provided by one incorporating the coefficients derived from the Cox model (Table XI).

For those patients who survived the first 6 months, disease stage, PS and Na appear to be the important prognostic factors with stage being of primary importance. In addition, AlkP, GGT and bicarbonate were also significant variables in some of the analyses. Since the predictive power of most variables decreased with increasing duration of follow-up, a prognostic index for this later period based on the values of disease stage, PS and Na (or other variables) measured at the start of treatment is not particularly useful. A more relevant index for this period would be one derived from the values of PS, stage, Na, etc., measured in 6 month survivors, together with other variables such as response to treatment during the previous months and tumour progression. The decreasing predictive power of prognostic factors over time is also illustrated by the work of Souhami and Law (1990). They analysed the survival of patients who were alive at 2 years in the studies included in this overview, together with 2 year survivors in some other SCLC studies. Disease stage and age did not have any predictive value for survival beyond 2 years.

The risk ratios for PS and stage for the patients who survived for 2 or more years appear to indicate that the patients with a PS score of 2 or 3 have a better prognosis than those with a score of 1 and that those with ED have a better prognosis than those with LD (Table VII). This is, of course, most unlikely and is probably due to random variation. A surprisingly high proportion (39%) of the 104 two year survivors had a PS score of 2 or 3, while 19% were recorded as having ED; even higher proportions were found in some of the individual data sets. The different performance status scales used in the studies, which led to the derivation of an overall PS scale, may have reduced the prognostic efficiency of this variable. However, it is also likely that the high (and varying) proportions of 2 year survivors with a PS score of 2 or 3 and with ED indicate that standard criteria for these two variables may not have been applied in all hospitals.

There was certainly some variation in the evaluation of disease stage in the individual studies (Allan *et al.*, 1984; Cerny *et al.*, 1987; Cullen *et al.*, 1986; MRC Lung Cancer Working Party, 1979, 1981, 1983; Smyth *et al.*, 1986; Souhami *et al.*, 1984, 1985; Vincent *et al.*, 1987). However, in general, the more complex tests and investigations, e.g. liver ultrasound scans, radioisotopic bone scans, bone marrow aspiration and CT scans, were only performed if there was clinical suspicion or 'plain' radiological evidence of extension beyond LD. Some of the investigations used to evaluate disease stage are time-consuming and unpleasant for the patient and, for this reason, Vincent *et al.* (1987) deliberately excluded stage from their attempt to devise a simple prognostic index. It should, nevertheless, be noted that there were important differences in survival between patients with LD and ED in their data. In an attempt to assess the value of disease stage, the nine analyses of the initial 6 month period (Tables IV and V) were re-performed omitting this variable. In the first analysis, it was found that a 'reduced' model consisting of PS and AlkP provided similar but not as good discrimination as that obtained with PS, AlkP and stage. However, unlike the original analyses which indicated quite consistently that PS, AlkP and stage were the most important variables, the results of the analyses omitting stage did not consistenly suggest a particular model that would act as a useful substitute. In all these analyses, the omission of stage reduced the degree of discrimination between the 'better' and 'medium' groups. Disease stage is the principal prognostic factor beyond 6 months and, therefore, this variable should not be omitted from any prognostic prediction for this period based on pretreatment measurements. Consequently, it is suggested that stage should continue to be assessed.

This overview has shown that, in the short-term (the 6 months after starting treatment), there are three groups of variables that are of importance for prognosis prediction. The first consists of PS, AlkP and disease stage which have been identified as being of primary importance. The second group comprises age, sex, Na, GGT, albumin, urea and chloride; these variables significantly improved the goodness of fit in the proportional hazards regression analyses, but the improvement in the prediction of survival obtained by their inclusion was small. LDH and AST form the third group; they are potentially important prognostic factors, but the relatively small numbers of patients for whom these measurements were available prevented any definitive conclusions about their role. In the longer term (6–24 months), stage, PS and Na are the significant variables, although stage appears to be the most important.

These results, therefore, led the Subcommittee to recommend that in future SCLC studies, performance status, disease stage, AlkP, Na, AST and LDH should be recorded; in addition, it is preferable that GGT, albumin, urea, plasma chloride and blood bicarbonate should also be measured. It should be stressed, however, that standard criteria must be applied meticulously when determing performance status and disease stage. The recording of the recommended measurements would allow standardised comparisons between new studies and possibly the selection of patients for different treatment strategies. It would also facilitate another overview of SCLC studies at some future date which should validate the present analysis and lead to more definite conclusions about prognostic factors for this disease.

### Appendix

*Activity status scale used in MRC1*

1. At work or active retirement
2. Full activity but not at work
3. Out and about but activity restricted
4. Confined at home or hospital
5. Bedridden

*Activity status scale used in MRC2*

1. Normal activity
2. Activity restricted
3. Confined to home/hospital
4. Bedridden

The Subcommittee is indebted to the following for allowing the use of their data: Professor N.M. Bleehan, MRC Cancer Trials Office, Cambridge; Dr M.H. Cullen, Queen Elizabeth Hospital, Birmingham; Dr D.J. Girling, MRC Cardiothoracic Epidemiology Group, London; Dr I.E. Smith, Royal Marsden Hospital, Sutton, Surrey; Professor J.F. Smyth, Western General Hospital, Edinburgh; Professor R.L. Souhami, University College Hospital and Middlesex School of Medicine, London; Dr N. Thatcher, Christie Hospital and Holt Radium Institute, Manchester. We also thank the following data managers and analysts for their assistance: Katy Ash, Sue Ashley, Gilly Coombes, Walter Gregory, Sharon Love, Richard Stephens, Moira Stewart, Ric Swindell and Eileen Williams. The Institute of Cancer Research receives support from the Cancer Research Campaign and the Medical Research Council.

### References

ALLAN, S.G., GREGOR, A., CORNBLEET, M.A. & 4 others (1984). Phase II trial of vindesine and VP16-213 in the palliation of poor prognosis patients and elderly patients with small cell lung cancer. *Cancer Chemother. Pharmacol.*, **13**, 106.

CERNY, T., BLAIR, V., ANDERSON, H., BRAMWELL, V. & THATCHER, N. (1987). Pre-treatment prognostic factors and scoring system in 407 small cell lung cancer patients. *Int. J. Cancer*, **39**, 146.

COX, D.R. (1972). Regression models and life tables (with discussion). *J. R. Stat. Soc. B*, **34**, 187.

CULLEN, M., MORGAN, D., GREGORY, W. & 27 others (1986). Maintenance chemotherapy for anaplastic small cell carcinoma of the bronchus: a randomised, controlled trial. *Cancer Chemother. Pharmacol.*, **17**, 157.

LEE, E.T. (1980). *Statistical Methods for Survival Data Analysis*. Lifetime Learning Publications: Belmont, California.

KARNOFSKY, D.A. & BURCHENAL, J.H. (1949). The clinical evaluation of chemotherapeutic agents in cancer. In *Evaluation of Chemotherapeutic Agents*, MacLeod, C.M. (ed.) p. 191. Columbia University Press: New York.

MEDICAL RESEARCH COUNCIL LUNG CANCER WORKING PARTY (1979). Radiotherapy alone or with chemotherapy in the treatment of small cell carcinoma of the lung. *Br. J. Cancer*, **40**, 1.

MEDICAL RESEARCH COUNCIL LUNG CANCER WORKING PARTY (1981). Radiotherapy alone or with chemotherapy in the treatment of small cell carcinoma of the lung: the results at 36 months. *Br. J. Cancer*, **44**, 611.

MEDICAL RESEARCH COUNCIL LUNG CANCER WORKING PARTY (1983). Cytotoxic chemotherapy before and after radiotherapy compared with radiotherapy followed by chemotherapy in the treatment of small cell carcinoma of the bronchus: the results up to 36 months. *Br. J. Cancer*, **48**, 755.

MEDICAL RESEARCH COUNCIL LUNG CANCER WORKING PARTY (1989*a*). Survival, adverse reactions and quality of life during combination chemotherapy compared with selective palliative treatment for small cell lung cancer. *Respir. Med.*, **83**, 51.

MEDICAL RESEARCH COUNCIL LUNG CANCER WORKING PARTY (1989*b*). Controlled trial of twelve versus six courses of chemotherapy in the treatment of small cell lung cancer. *Br. J. Cancer*, **59**, 584.

MORITTU, L., EARL, H.M., SOUHAMI, R.L. & 5 others (1989). Patients at risk of chemotherapy-associated toxicity in small cell lung cancer. *Br. J. Cancer*, **59**, 801.

SMYTH, J.F., FOWLIE, S.M., GREGOR, A. & 4 others (1986). The impact of chemotherapy on small cell carcinoma of the bronchus. *Q. J. Med.*, **61**, 969.

SOUHAMI, R.L., GEDDES, D.M., SPIRO, S.G. & 5 others (1984). Radiotherapy in small cell cancer of the lung treated with combination chemotherapy: a controlled trial. *Br. Med J.*, **288**, 1643.

SOUHAMI, R.L., BRADBURY, I., GEDDES, D.M., SPIRO, S.G., HARPER, P.G. & TOBIAS, J.S. (1985). Prognostic significance of laboratory parameters measured at diagnosis in small cell carcinoma of the lung. *Cancer Res.*, **45**, 2878.

SOUHAMI, R.L. & LAW, K. (1990). The prognosis of small cell lung cancer. *Br. J. Cancer* (in the press).

SPIRO, S.G., SOUHAMI, R.L., GEDDES, D.M. & 6 others (1989). Duration of chemotherapy in small cell lung cancer: a Cancer Research Campaign trial. *Br. J. Cancer*, **59**, 578.

THATCHER, N., BARBER, P.V., HUNTER, R.D. & 4 others (1982). Eleven week course of sequential methotrexate, thoracic irradiation, and moderate-dose cyclophosphamide for 'limited' stage small cell bronchogenic carcinoma: a study from the Manchester Lung Tumour Group. *Lancet*, **i**, 1040.

THATCHER, N., CERNY, T., STOUT, R. & 5 others (1987). Ifosfamide, etoposide, and thoracic irradiation therapy in 163 patients with unresectable small cell lung cancer. *Cancer*, **60**, 2382.

THATCHER, N., JAMES, R.D., STEWARD, W.P. & 4 others (1985*a*). Three months treatment with cyclophosphamide VP-16-213 followed by methotrexate and thoracic radiotherapy for small cell lung cancer. *Cancer*, **56**, 1332.

THATCHER, N., STOUT, R., SMITH, D.B. & 4 others (1985*b*). Three months treatment with chemotherapy and radiotherapy for small cell lung cancer. *Br. J. Cancer*, **52**, 327.

VINCENT, M.D., ASHLEY, S.E. & SMITH, I.E. (1987). Prognostic factors in small cell lung cancer: a simple prognostic index is better than conventional staging. *Eur. J. Cancer Clin. Oncol.*, **23**, 1589.

WORLD HEALTH ORGANIZATION (1979). *WHO Handbook for Reporting Results of Cancer Treatment* (WHO Offset Publication No. 48). World Health Organization: Geneva.

ZUBROD, C.G., SCHNEIDERMAN, M., FREI, E. III & 16 others (1960). Appraisal of methods for the study of chemotherapy of cancer in man: comparative therapeutic trial of nitrogen mustard and thriethylene thiophosphoramide. *J. Chron. Dis.*, **11**, 7.