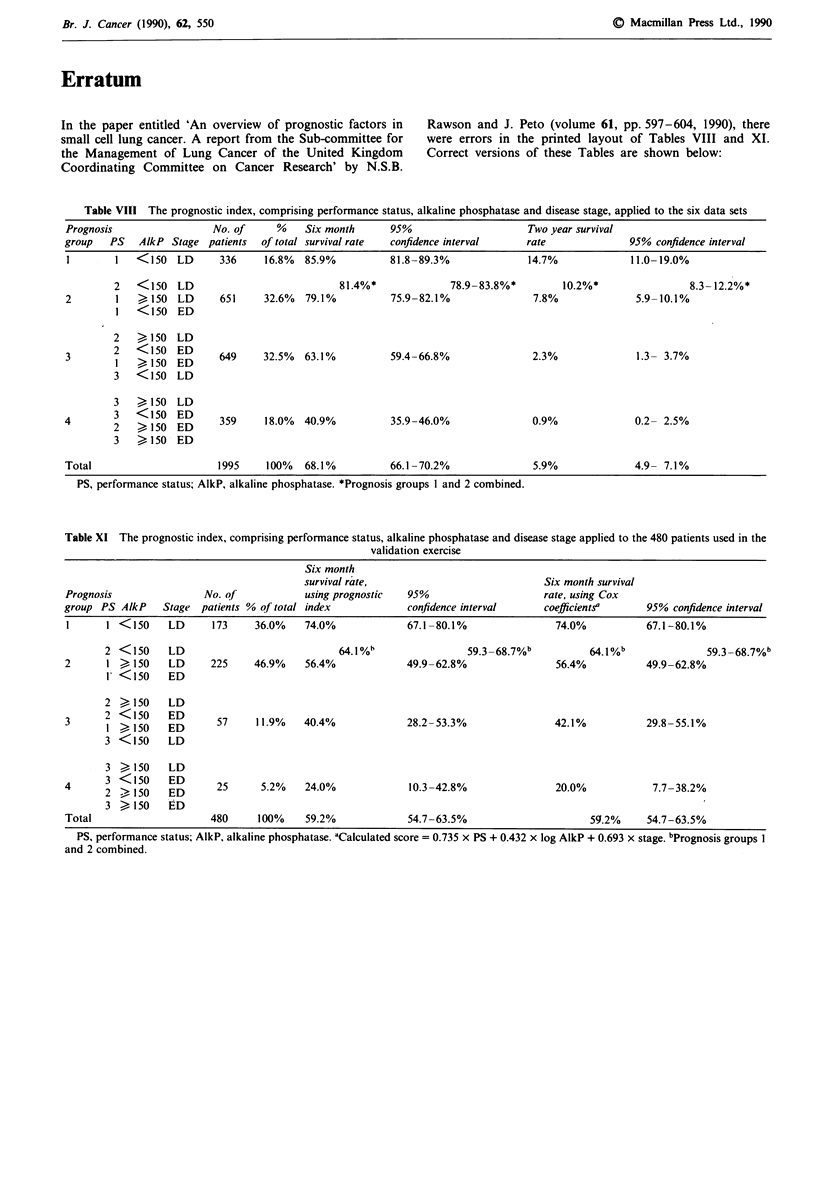# Erratum

**Published:** 1990-09

**Authors:** 


 © Macmillan Press Ltd., 1990

### Erratum

In the paper entitled 'An overview of prognostic factors in small cell lung cancer. A report from the Sub-committee for the Management of Lung Cancer of the United Kingdom Coordinating Committee on Cancer Research' by N.S.B. Rawson and J. Peto (volume **61**, pp. 597–604, 1990), there were errors in the printed layout of Tables VIII and XI. Correct versions of these Tables are shown below:

**Table VIII** The prognostic index, comprising performance status, alkaline phosphatase and disease stage, applied to the six data sets

| *Prognosis group* | *PS* | *AlkP* | *Stage* | *No. of patients* | *% of total* | *Six month survival rate* | *95% confidence interval* | *Two year survival rate* | *95% confidence interval* |
|---|---|---|---|---|---|---|---|---|---|
| 1 | 1 | <150 | LD | 336 | 16.8% | 85.9% | 81.8–89.3% | 14.7% | 11.0–19.0% |
| | 2 | <150 | LD | | | 81.4%* | 78.9–83.8%* | 10.2%* | 8.3–12.2%* |
| 2 | 1 | ≥150 | LD | 651 | 32.6% | 79.1% | 75.9–82.1% | 7.8% | 5.9–10.1% |
| | 1 | <150 | ED | | | | | | |
| | 2 | ≥150 | LD | | | | | | |
| | 2 | <150 | ED | | | | | | |
| 3 | 1 | ≥150 | ED | 649 | 32.5% | 63.1% | 59.4–66.8% | 2.3% | 1.3– 3.7% |
| | 3 | <150 | LD | | | | | | |
| | 3 | ≥150 | LD | | | | | | |
| | 3 | <150 | ED | | | | | | |
| 4 | 2 | ≥150 | ED | 359 | 18.0% | 40.9% | 35.9–46.0% | 0.9% | 0.2– 2.5% |
| | 3 | ≥150 | ED | | | | | | |
| Total | | | | 1995 | 100% | 68.1% | 66.1–70.2% | 5.9% | 4.9– 7.1% |

PS, performance status; AlkP, alkaline phosphatase. *Prognosis groups 1 and 2 combined.

**Table XI** The prognostic index, comprising performance status, alkaline phosphatase and disease stage applied to the 480 patients used in the validation exercise

| *Prognosis group* | *PS* | *AlkP* | *Stage* | *No. of patients* | *% of total* | *Six month survival rate, using prognostic index* | *95% confidence interval* | *Six month survival rate, using Cox coefficients*[a] | *95% confidence interval* |
|---|---|---|---|---|---|---|---|---|---|
| 1 | 1 | <150 | LD | 173 | 36.0% | 74.0% | 67.1–80.1% | 74.0% | 67.1–80.1% |
| | 2 | <150 | LD | | | 64.1%[b] | 59.3–68.7%[b] | 64.1%[b] | 59.3–68.7%[b] |
| 2 | 1 | ≥150 | LD | 225 | 46.9% | 56.4% | 49.9–62.8% | 56.4% | 49.9–62.8% |
| | 1 | <150 | ED | | | | | | |
| | 2 | ≥150 | LD | | | | | | |
| | 2 | <150 | ED | | | | | | |
| 3 | 1 | ≥150 | ED | 57 | 11.9% | 40.4% | 28.2–53.3% | 42.1% | 29.8–55.1% |
| | 3 | <150 | LD | | | | | | |
| | 3 | ≥150 | LD | | | | | | |
| | 3 | <150 | ED | | | | | | |
| 4 | 2 | ≥150 | ED | 25 | 5.2% | 24.0% | 10.3–42.8% | 20.0% | 7.7–38.2% |
| | 3 | ≥150 | ED | | | | | | |
| Total | | | | 480 | 100% | 59.2% | 54.7–63.5% | 59.2% | 54.7–63.5% |

PS, performance status; AlkP, alkaline phosphatase. [a]Calculated score = 0.735 × PS + 0.432 × log AlkP + 0.693 × stage. [b]Prognosis groups 1 and 2 combined.